# Sown Summer-Blooming Wildflowers as a Tool to Support Pollinator Biodiversity During Dry Periods in Mediterranean Agroecosystems

**DOI:** 10.3390/plants15060887

**Published:** 2026-03-12

**Authors:** Stefano Benvenuti

**Affiliations:** Department of Agri-Food Sciences and Technologies, University of Bologna, Viale Giuseppe Fanin 46, 40127 Bologna, Italy; stefano.benvenuti8@unibo.it

**Keywords:** plant–insect mutualism, biodiversity restoration, wildflower strip, resilience to climate change, sustainability

## Abstract

Summer abiotic stresses typical of Mediterranean agro-environments, now exacerbated by climate change, reduce floral resource availability and further compromise the survival of pollinators already threatened in the so-called Anthropocene. The aim of this study was to evaluate several summer-blooming wildflower species, collected from ecologically disturbed and diversified habitats, in order to assess their ecological role in attracting pollinators within agroecosystems. The primary dormancy typical of wild species seeds was partially overcome through appropriate pre-sowing seed treatments, while secondary dormancy was reduced by soil rolling after sowing. Soil rolling proved particularly beneficial for species with very small seeds, highlighting the importance of adequate seed–soil contact for successful establishment. All tested species exhibited summer flowering between May and July, with some flowering later in the season, and showed high attractiveness to pollinators both in terms of abundance and taxonomic diversity. However, this ecosystem service declined significantly in the second year, although certain species demonstrated a strong capacity to persist and to maintain satisfactory pollinator attractiveness over time. In conclusion, while the experiment revealed several critical aspects, it also provides encouraging prospects for further research aimed at enhancing pollinator survival in agroecosystems that are severely threatened by pollinator decline.

## 1. Introduction

One of the major environmental challenges of the new millennium is the progressive decline in pollinators [1] and the consequent loss of the ecosystem services they provide [2]. This threat is not only environmental but also economic [3], as the productivity of many entomophilous crops [4] relies heavily on pollinator activity, thereby raising serious concerns for food security [5,6].

Pollinator decline results from a wide range of drivers, often related to agricultural practices. These include not only the use of pesticides [7], but also the progressive simplification of cropping systems [8], characterized by increasingly short crop rotations and the reduction or loss of insect-pollinated forage crops, particularly those belonging to the highly valuable Fabaceae family [9]. In addition to the erosion of crops rich in pollen and nectar, there is also a marked decline in insect-pollinated weeds which, despite representing an agronomic constraint, historically contributed to sustaining pollinator populations [10]. Habitat quality could be improved through effective monitoring with target-oriented indicators and by incentivising collaboration among land managers to support spatially targeted management [11]. Even flowering plants in urban gardens, embedded within the landscape mosaic, may contribute to the persistence of pollinator biodiversity [12,13].

The economic constraints, or even the impracticality, of reintroducing cropping systems that predate the so-called “Green Revolution” necessitate the development of agronomic strategies capable of supporting the survival of a wide range of pollinating insects. In particular, the inability to revert to pre-Green Revolution agricultural systems [14] highlight the need for practices that ensure prolonged flowering periods within agroecosystems [15]. For this reason, especially in highly industrialized countries dominated by conventional cropping systems [16], wildflower strips have been established along self- or wind-pollinated arable crops [17] or orchards [18]. The plant species selected for these multifunctional strips [19] are typically wild taxa [20,21], often collected from the surrounding local areas [22,23] or, at a minimum, native to the target environment [24], and may also include insect-pollinated weed species. Among these, the most suitable species for agronomic use are wildflower weeds that have persisted mainly in hilly and/or mountainous agroecological refugia, where traditional cropping systems have been maintained [25]. However, the limited availability of floral resources during the summer months, particularly in Mediterranean environments characterized by high temperatures and drought, conditions frequently exacerbated by climate change [26], remains a major challenge. Consequently, ensuring pollinator survival during the summer period within agroecosystems is of critical importance. In this context, several herbaceous species adapted to heat and water stress are widely distributed across Mediterranean landscapes, largely as a result of their pronounced spatial heterogeneity [27]. Consequently, the use of these species in the design of summer-flowering wildflower strips may be of primary importance. However, their agronomic management remains poorly understood. Although several studies have investigated their germination ecology under laboratory conditions [28], information on germination and seedling emergence following field sowing is still scarce, particularly in Mediterranean environments [29]. In addition, their agronomic use is constrained not only by the presence of primary seed dormancy, which is typical of many wild species [30], but also by soil-mediated inhibitory effects after sowing, which may induce secondary dormancy [31]. These constraints are exacerbated in clay-textured soils, especially for species with very small seeds [32], which require highly accurate seedbed preparation [33].

A further challenge arises from competition with pre-existing weeds originating from soil seed banks [34]. Moreover, the temporal sustainability of wildflower strips, often limited to a short-duration in the time [35], is compromised by the poor self-reseeding capacity of many wildflower species [36] and depends largely on the resilience of perennial species [37].

The objectives of this experiment were as follows: (i) to evaluate the performance of sown summer-flowering wildflower strips; (ii) to quantify their attractiveness to pollinators and assess the associated level of pollinator biodiversity; and (iii) to examine the temporal sustainability of each wildflower species during the second year after sowing.

## 2. Materials and Methods

### 2.1. Plant Material

**Figure 1:**
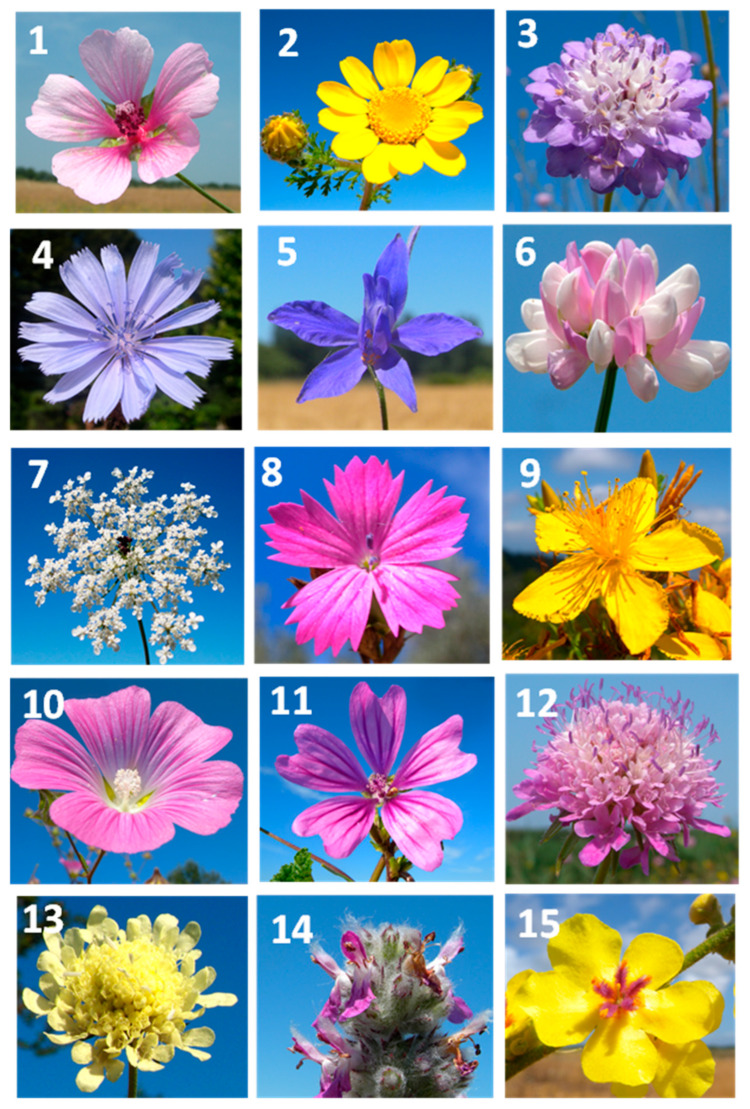
Flowers of the fifteen tested wildflowers species: (**1**) = *A. cannabina*. (**2**) = *A. radiatus*. (**3**) = *C. transsylvanica*. (**4**) = *C. inthybus*. (**5**) = *C. regalis*. (**6**) = *C. varia*. (**7**) = *D. carota*. (**8**) = *D. carthusianorum*. (**9**) = *H. perforatum*. (**10**) = *L. punctata*. (**11**) = *M. sylvestris*. (**12**) = *S. columbaria*. (**13**) = *S. ochroleuca*. (**14**) = *S. germanica*. (**15**) = *V. sinuatum*.

Fifteen herbaceous wild species (Table 1) were selected for this study based on three main criteria: (i) their widespread occurrence within and/or in the surroundings of different agroecosystems; (ii) the evident evolutionary development of their flowers toward pollinator attraction; and (iii) their flowering period occurring also, or exclusively, during the hottest midsummer months. The showy corollas of all fifteen wildflower species are illustrated in Figure 1.

**Figure 2:**
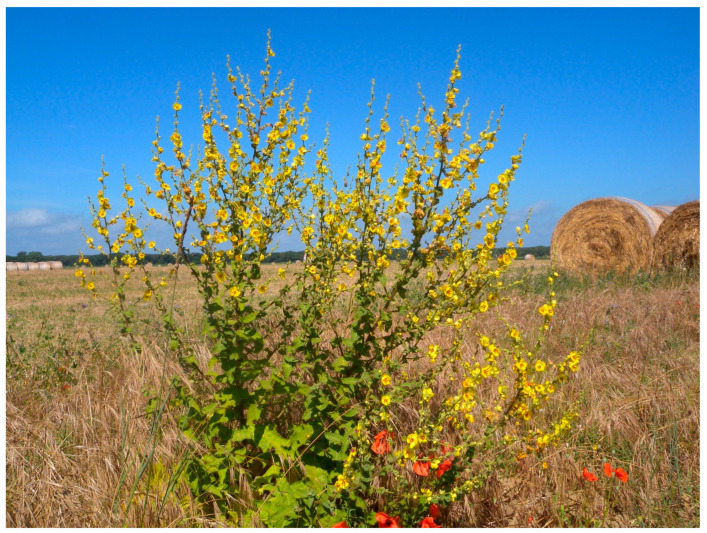
Example of environment of seed collection of one of the fifteen wildflower species studied (in this case *Verbascum sinuatum*) where the summer aridity typical of the Mediterranean environment is evident.

In early autumn 2021, senescent flowers or inflorescences of each selected species were collected from various Mediterranean agro-environments (Figure 2) specified in the above-mentioned Table 1. Seeds were extracted in the laboratory from the corresponding senescent floral tissues, then cleaned, dried to a maximum moisture content of 12%, and stored in glass jars at 20 °C under 50% relative air humidity.

### 2.2. Agronomic Environment

The trials were conducted in the experimental fields of the Department of Agriculture, Food and Environment at the University of Pisa (43°40′39″ N, 10°19′46″ E), Italy. The soil was classified as a Xerofluvent with a sandy loam texture, characterized by 70% sand, 18% silt, and 12% clay, a pH of 7.5, and an organic matter content of 1.8%. The climate of this Mediterranean environment is characterized by an average annual rainfall of approximately 800 mm, mainly concentrated in autumn and spring, and by typically hot and dry summers. Mean minimum and maximum daily air temperatures during the spring–summer period range as follows: May, 14/24 °C; June, 18/28 °C; July, 20/30 °C; August, 20/30 °C; and September, 16/26 °C (minimum/maximum, respectively).

Rectangular plots (2 × 10 m) were established in an area adjacent to maize fields in order to minimize potential interference from pollinators associated with insect-pollinated crops.

### 2.3. Laboratory Evaluation of Seed Germination and Treatments to Remove Dormancy

Seeds of all species were subjected to germination tests. For each species, seeds were imbibed on a single sheet of moistened filter paper (Whatman No. 1; 7 mL of distilled water) and placed in 12 cm diameter Petri dishes (50 seeds per dish). Incubation was carried out in climate-controlled growth chambers under alternating temperatures of 15/25 °C, corresponding to dark/light conditions, respectively, with a 12 h/12 h photoperiod. Illumination (approximately 50 μmol m^−2^ s^−1^) was provided by fluorescent lamps (Philips THL 20W/33, Eindhoven, The Netherlands).

Germination was recorded daily, and seeds were considered germinated upon radicle emergence, reaching a length of 2 mm. Germination trials were terminated when final germination percentages were attained for each species, defined as the absence of further germination for at least one week.

Following these preliminary tests, seeds of each species were subjected to different dormancy-breaking treatments (scarification, washing, or chilling) selected on the basis of previous studies [38]. The seed treatment protocols were as follows: (i) mechanical scarification by gently rubbing seeds with sandpaper for approximately 1–2 min; (ii) seed washing in running water for 12 h; and (iii) cold stratification (chilling) in moistened Petri dishes at 4 °C for 2 weeks. All species were subjected to each treatment; however, only those treatments that resulted in the highest dormancy-breaking effectiveness are reported in this study.

### 2.4. Seed Weight

Seed weight (or fruit weight, in the case of achenes from Asteraceae, Dipsacaceae, and Apiaceae) was determined by weighing 1000 randomly selected seeds in accordance with the International Seed Testing Association (ISTA) rules for seed testing [39].

### 2.5. Preliminary Stale Seedbed Preparation on Experimental Plots

In October 2021, March 2022, and July 2022, three “stale seedbed preparation” interventions were carried out to reduce the soil seed bank in each selected experimental plot. This preventive agronomic strategy, designed to induce the germination of buried seeds, consisted of two rotary harrow (about 10 cm deep) passes (one in autumn, one in the following spring and the last one in the subsequent late summer) applied to each of the fifteen experimental plots. This management practice aimed to minimize the unavoidable interference between the experimental wildflowers and pre-existing weed species.

### 2.6. Preliminary Seedling Emergence Test of Treated Seeds with or Without Soil Rolling

To obtain a uniform plant density, treated seeds were tested under field conditions using real soil sowing. The experiment was conducted in October 2021. For each plant species, the seed dose and the seedling emergence dynamics of the treated seeds were evaluated. Sowing was performed using the treated seeds that had shown the best germination performance in laboratory in vitro tests for each wildflower species.

Seeds were sown manually at a density of 500 seeds m^−2^, calculated on the basis of the thousand-seed weight of each species, in order to subsequently evaluate the emergence rate (number of emerged seedlings per number of seeds sown). Each experimental plot (2 × 10 m) for the fifteen plant species (15 species × 3 replicates, total 45 plots) was divided into two subplots to assess the effect of soil rolling. Soil rolling was applied only to one half of each plot using a manual roller (68 kg, 0.5 m wide).

After manual sowing, soil rolling was carried out on half of the plotsafter used to evaluate pollinator attractiveness. This treatment was applied to assess the effect of this agronomic practice on soil–seed contact and, consequently, on seedling emergence dynamics.

Seedling emergence dynamics of all wildflower species were assessed two months after sowing (December 2021) by placing ten square metal frames (0.5 × 0.5 m) per subplot on the experimental plots, both with and without soil rolling.

### 2.7. Wildflower Field Sowing

In the first ten days of October 2022, following preliminary seed germination and seedling emergence tests, sowing of fifteen wildflower species was carried out based on the actual seedling emergence rate of each species. To achieve a uniform plant density, the sowing rate for each species was calculated by taking into account the 1000-seed weight (proportionally) and the emergence rate of treated seeds (inversely). Seedbed preparation consisted of two successive passes with a rotary harrow to create fine soil aggregates, thereby optimizing the germination of small seeds, as reported in previous studies [40]. Immediately after sowing, soil rolling was performed. The target overall wildflower density was 100 plants m^−2^. Each species was sown individually within each experimental plot in order to better assess both emergence dynamics and subsequent pollinator visitation. The experimental design was a randomized complete block with three replicates per species (15 species × 3 replicates, for a total of 45 plots). Mean air temperatures during October, November and December 2023 were 16.5 °C, 11.9 °C and 8.0 °C, respectively.

### 2.8. Flowering Dynamics

For each of the fifteen wildflower species, ten randomly selected individuals were tagged with paper labels to monitor flowering dynamics over time (from May to September). Flowering data were recorded as the onset (first open flower) and end of the flowering period throughout the experimental duration. Observations for each wildflower species were collected twice a week. Flowering dynamics were also assessed during the second and third year; however, since no statistically significant differences were detected between the two experimental years, data from both years (2023 and 2024) were pooled for analysis.

### 2.9. Evaluation of Flower Visitors of the First Experimental Year

Pollinator activity—defined as insects repeatedly visiting flowers with clear flower–pollinator contact (with evident food collection)—was assessed during the flowering periods (May–September) of both experimental years (2023 and 2024). The climatic parameters recorded during the experimental biennium were consistent with the above-mentioned long-term climatic averages. Data were collected using a 1 m^2^ plastic frame placed at the center of each plot. Pollinators landing on flowers within this area and showing evident pollen and/or nectar foraging behavior were observed, identified (within the taxonomic categories listed below), and counted. Pollinators were classified into the following groups: honey bees (*Apis mellifera*), solitary bees, bumblebees, Diptera (Syrphidae, Bombyliidae, or Tachinidae), Lepidoptera, and Coleoptera.

Observations were conducted weekly throughout the entire flowering period during the morning hours (07:00–11:00 h), as pollinator visitation rates decline during warmer periods of the day (personal observation). Each observation was performed for each of the 45 experimental plots (15 wildflower species × 3 replicates). Observations were conducted from a distance of at least one meter in order to avoid interference with pollinator visitation dynamics. Pollinator visitation was expressed as flower visits m^−2^ h^−1^ for each wildflower species, and the relative proportion of each pollinator group was calculated.

Pollinator visits were recorded for all wildflower species in both years (2023 and 2024). However, data from the second year were expressed as the reduction in flower visits, reflecting the expected decrease in plant density.

### 2.10. Agronomic Management After the First Experimental Year

After reaching the phenological stage of complete senescence (October 2023), each wildflower plot was subjected to vegetation shredding. The senescent wildflower plots were shredded using a standard on-farm shredder with the plant residues left in the field. This agronomic disturbance was implemented to assess the re-colonization capacity of the different wildflower species and to evaluate their survival and/or self-seeding potential in the second year, as well as the persistence of the ecosystem services provided by pollinators.

### 2.11. Evaluation of Plant Density in the Second Experimental Year

During the winter following vegetation shredding (January 2024), the density of disturbance-resilient seedlings (perennials) and self-sown seedlings (annuals and perennials) was evaluated. The plant density of each plant species was evaluated in spring (April 2024) by placing the above cited square metal frame (0.5 m) in each experimental plot.

### 2.12. Calculation of Diversity Index of Pollinator Communities

Data of the pollinators of both experimental years (2023–2024) were used to calculate the Shannon diversity index (*H*′) as follows:

k *H*′ = −∑p*_i_* log p*_i_* I = 1

where k is the number of plant or pollinator species, and p*_i_* is the fraction of individuals belonging to the *i*-th each wildflower community or pollinator species. Biodiversity indices of pollinator visits in the second year were shown to decline compared to the previous year following the inevitable and expected decline in the sustainability of blooms over time.

### 2.13. Statistical Analyses

All experiments were conducted using a randomized complete block design with three replicates. After testing for homogeneity of variances, percentage data were subjected to an arcsine square-root transformation to normalize their distribution [41]. Both transformed percentage data and untransformed non-percentage data were analyzed by analysis of variance (ANOVA). Mean separation was performed using Duncan’s Multiple Range Test at *p* < 0.05 and/or *p* < 0.01.

The variables analyzed about seed germination and seedling emergence were as follows: (i) wildflower seed treatment; (ii) soil rolling after sowing. The other variables analyzed about pollinators were as follows: (i) total number of visitors (visits m^−2^ h^−1^); (ii) pollinator biodiversity across different functional groups (honey bees, other bees, bumblebees, Diptera—Syrphidae, Bombyliidae, or Tachinidae—Lepidoptera, and Coleoptera); and (iii) plant density and biodiversity loss in the second year.

In addition, linear regressions were performed between the 1000-seed weight of the fifteen wildflower species and the relative percentage improvement in seedling emergence attributable to soil rolling. All statistical analyses were carried out using CoHort software 6.4 (Minneapolis, MN, USA).

## 3. Results

### 3.1. Seed Germination and Dormancy-Breaking Treatments

All species investigated exhibited seed dormancy (Table 2). None of the fifteen species reached 50% germination when incubated without any pre-treatment. The degree of dormancy was strongly species-dependent, ranging from 15.5% in *Consolida regalis* to 48.1% in *Cephalaria transsylvanica*. However, dormancy was consistently alleviated by appropriate seed treatments, all of which resulted in statistically significant improvements (*p* < 0.01 or *p* < 0.05).

Seed scarification was the most effective treatment for the three species belonging to the family Malvaceae (*Althaea cannabina*, *Lavatera punctata*, and *Malva sylvestris*), with post-treatment germination rates of 76.5%, 88.2%, and 92.2%, respectively, as well as for *Coronilla varia* (88.5%) of the family Fabaceae. Seed washing was effective in three Dipsacaceae species (*Cephalaria transsylvanica*, *Scabiosa columbaria*, and *Scabiosa ochroleuca*), resulting in germination rates of 75.6%, 77.3%, and 75.5%, respectively, and in two Asteraceae species (*Anacyclus radiatus* and *Cichorium intybus*), with germination rates of 52.3% and 64.2%, respectively.

Finally, cold stratification (chilling) effectively promoted germination in *Consolida regalis* (54.5%), *Daucus carota* (65.4%), *Dianthus carthusianorum* (72.2%), *Stachys germanica* (66.2%), and *Verbascum blattaria* (51.5%), belonging to the families Ranunculaceae, Apiaceae, Caryophyllaceae, Lamiaceae, and Scrophulariaceae, respectively.

### 3.2. Seedling Emergence Test

Despite the aforementioned dormancy removal, sowing in a soil matrix reduced seed germination and seedling emergence (Table 3). With the exception of *M. sylvestris*, which reached a seedling emergence of 53.7%, all other species showed emergence values below 50% when soil rolling was not applied after sowing. In particular, *Anacyclus radiatus* and *Verbascum sinuatum* exhibited seedling emergence below 20%.

**Figure 3:**
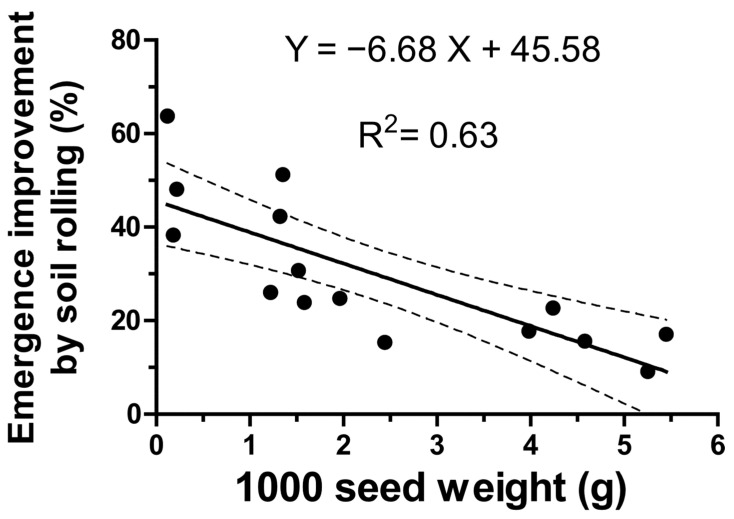
Linear regression between 1000 seed weight (g) of the fifteen wildflower species and the relative seedling emergence improvement percentage due to soil rolling. The relative equation (significant at *p* < 0.05), the R^2^ values and the 95% confidence levels are reported.

However, soil rolling after sowing significantly increased seedling emergence dynamics across species (*p* < 0.01 or *p* < 0.05). The most pronounced effects (*p* < 0.01) were observed in *A. radiatus*, *Dianthus cartusianorum*, *Hypericum perforatum*, *Stachys germanica*, and *Verbascum sinuatum*. The positive effect of soil rolling was closely associated with seed unit weight. Linear regression analysis between the percentage increase in seedling emergence induced by soil rolling and the 1000-seed weight revealed a significant correlation (*p* < 0.05) and a relatively high R^2^ value (Figure 3).

### 3.3. Wildflower Flowering Dynamics

As expected, the flowering period of the fifteen species started in May and extended over the subsequent two months (Table 4). Approximately half of the tested species completed flowering by August. In contrast, the three species belonging to the family Dipsacaceae (*Centaurea transsylvanica*, *Scabiosa columbaria*, and *S. ochroleuca*), together with *Daucus carota* (Apiaceae), continued flowering into September. Finally, two of the three Malvaceae species (*Althaea cannabina* and *Malva sylvestris*) persisted in flowering until October.

### 3.4. Pollinator Flower-Visit Quantity and Biodiversity During the First Experimental Year

As expected, June and July showed the highest visitation rates (Table 5), with 40.7 and 46.2 visits m^−2^ h^−1^, respectively. Visitation significantly decreased (*p* < 0.05) in the following month, August (28.0 visits m^−2^ h^−1^). Although quantitatively lower, the early (May) and late (September) sampling periods also exhibited satisfactory visitation rates (9.8 and 18.7 visits m^−2^ h^−1^, respectively), indicating a prolonged period of pollinator activity.

Considering the entire experimental period (May–September), the two species belonging to the Dipsacaceae family (*C. transsylvanica* and *S. columbaria*) showed the highest visitation rates (240.0 and 187.7 visits m^−2^ h^−1^, respectively). High pollinator attractiveness was also observed in another Dipsacaceae species (*S. ochroleuca*, 156.3 visits m^−2^ h^−1^), two Malvaceae species (*M. sylvestris* and *A. cannabina*, 164.2 and 153.1 visits m^−2^ h^−1^, respectively), and *Daucus carota* (Apiaceae, 161.2 visits m^−2^ h^−1^). All remaining species exhibited lower, yet still satisfactory, attractiveness to pollinators, as visitation rates consistently exceeded 100 visits m^−2^ h^−1^.

**Figure 4:**
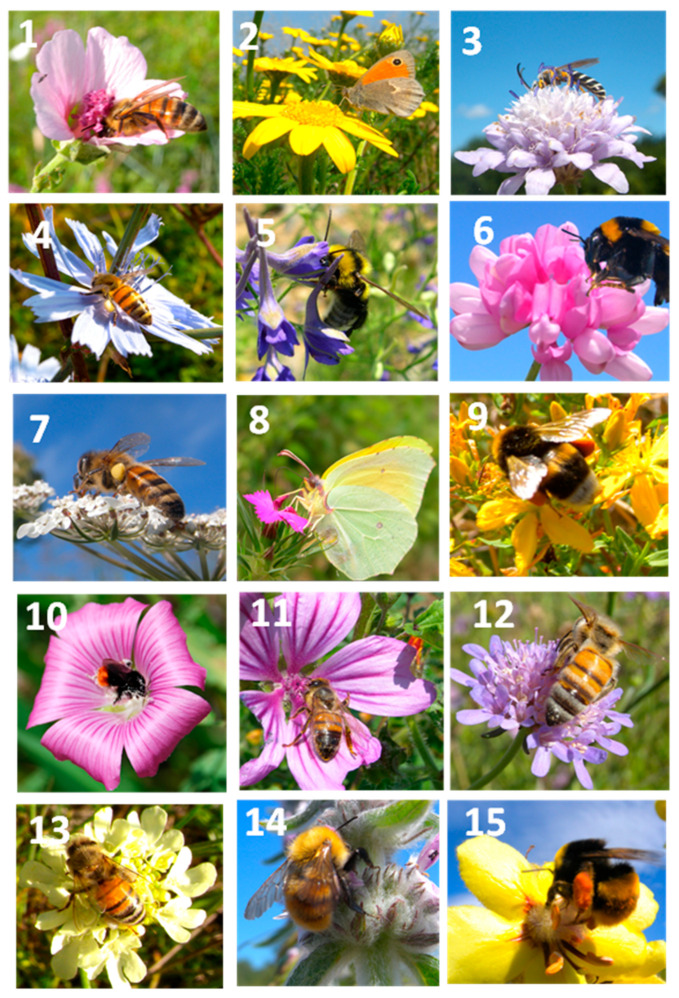
Categories of pollinators primarily observed on each of the fifteen wildflower species: (**1**) = Honeybee of *A. cannabina*. (**2**) = Lepidoptera on *A. radiatus*. (**3**) = Solitary bee on *C. transsylvanica*. (**4**) = Honeybee on *C. inthybus*. (**5**) = Bumblebee on *C. regalis*. (**6**) = Bumblebee on *C. varia*. (**7**) = Honeybee on *D. carota*. (**8**) = Lepidoptera on *D. carthusianorum*. (**9**) = Bumblebee of *H. perforatum*. (**10**) = Bumblebee on *L. punctata*. (**11**) = Honeybee on *M. sylvestris*. (**12**) = Honeybee on *S. columbaria*. (**13**) = Honeybee on *S. ochroleuca*. (**14**) = Bumblebee on *S. germanica*. (**15**) = Bumblebee on *V. sinuatum*.

The fifteen wildflower species differed in their attractiveness to the eight pollinator categories considered (Table 6). Honey bees were the predominant visitors of *C. intybus*, *A. cannabina*, *S. columbaria*, and *D. carota*, accounting for 52.8%, 45.3%, 37.4%, and 35.7% of total visits, respectively. In contrast, solitary bees were the main visitors to *C. transsylvanica*, *H. perforatum*, *M. sylvestris*, and *S. columbaria* (41.5%, 41.2%, 32.8%, and 32.4% of visits, respectively). Bumblebees were predominant on *V. sinuatum* and *S. germanica* (41.2% and 40.4%, respectively), whereas syrphid flies were the most frequent visitors—though less consistently—to *S. ochroleuca* and *D. cartusianorum* (14.2% and 13.2%, respectively). Other dipteran groups (Bombyliidae and Tachinidae) and beetles were less active, never exceeding 10% of total visits. Conversely, Lepidoptera, although generally infrequent across most species, were markedly predominant on *D. cartusianorum* (44.3%) and, to a lesser extent, on *A. radiatus* (26.4%). Figure 4 illustrates some of the most frequent pollinator visits recorded for each of the fifteen wildflower species.

The aforementioned dynamics of flower visitation across the fifteen plant species resulted in different levels of biodiversity, expressed as the Shannon diversity index (H′) for the various pollinator categories (Table 7). The highest Shannon index was observed for *H. perforatum* (0.82), which was statistically different from all other species (*p* < 0.05). High values (H′ > 0.7) were also recorded for *L. punctata*, *A. radiatus*, *D. carota*, and *S. ochroleuca*. All remaining plant species exhibited Shannon index values below 0.7. The lowest levels of pollinator biodiversity were calculated for the zygomorphic flowers of *C. regalis* (0.51), *S. germanica* (0.52), and *D. cartusianorum* (0.53).

### 3.5. Wildflower Survival and Pollinator Biodiversity During the Second Experimental Year

As expected, all wildflower species in their second year exhibited lower plant densities than in the previous year (Table 8). However, the magnitude of this reduction varied markedly among species. *M. sylvestris*, *D. carota*, and *S. columbaria* showed the smallest declines, maintaining 92.2%, 87.5%, and 84.8% of the previous year’s plant density, respectively. A lower but still satisfactory residual density was observed for *C. inhybus*, *D. cartusianorum*, *C. varia*, and *S. ochroleuca*, with residual densities of 87.5%, 68.9%, and 67.3%. All remaining species experienced a more pronounced decrease in plant density, falling below 60%. The lowest residual densities were recorded for *C. regalis* and *V. sinuatum* (24.0% and 34.6%, respectively).

This self-thinning of vegetation inevitably resulted in a reduction in flower visitation rates. The largest declines were observed for *V. sinuatum*, *A. radiatus*, and *C. regalis* (78.5%, 66.2%, and 61.2%, respectively), which were among the most strongly self-thinned species. Conversely, more resilient species (*M. sylvestris*, *S. columbaria*, *A. cannabina*, and *D. carota*) largely maintained their flower visitation rates, showing reductions of only 5.5%, 8.5%, 14.6%, and 14.4%, respectively. In contrast, pollinator biodiversity during the second experimental year remained largely unchanged, with only a slight, non-significant decrease in the Shannon diversity index (H′).

## 4. Discussion

### 4.1. Wildflower Strips Establishment

As expected, the selected plant species exhibited pronounced seed dormancy. Seed dormancy plays a crucial role in the survival dynamics of many species [42], as it prevents synchronized germination that could hinder recolonization following biotic or abiotic disturbances. Nevertheless, advances in the understanding of the physical, chemical, and/or physiological mechanisms underlying dormancy [43] have enabled successful germination through specific seed pre-treatments, including scarification, washing, and chilling.

The wildflower species belonging to the families Malvaceae (*A. cannabina*, *L. punctata*, and *M. sylvestris*) and Fabaceae (*C. varia*) were confirmed to possess seeds characterized by physical dormancy [44], which was effectively removed by scarification. In contrast, the remaining wildflower species exhibited chemical dormancy [35], which could be overcome by seed chilling (*C. regalis*, *D. carthusianorum*, *H. perforatum*, *S. germanica*, and *V. sinuatum*) or seed washing (*A. radiatus*, *C. transsylvanica*, *C. intybus*, *D. carota*, *S. columbaria*, and *S. ochroleuca*).

These findings indicate that the use of these species without appropriate seed pre-treatments results in poor agronomic performance due to unsatisfactory germination following direct field sowing. Although the applied treatments consistently promoted dormancy release under laboratory conditions (Petri dishes), the incubation of treated seeds in the soil matrix after field sowing substantially reduced the germination success mentioned above in vitro. This reduction in soil germination, observed in both crops [36] and weeds [45], appears to be associated with suboptimal seed–soil contact, which limits seed imbibition.

Indeed, soil rolling after sowing markedly enhanced seed germination by improving seed–soil contact. In this context, it has been demonstrated that the degree of contact increases as soil macroporosity decreases [46]. This beneficial effect of reduced macroporosity is particularly pronounced in species with small seeds. Accordingly, an inverse relationship was observed between the increase in germination following rolling and the weight of 1000 seeds. The effectiveness of rolling in promoting the germination of small-seeded species is especially important in areas with limited water availability [47], such as Mediterranean environments, where water uptake preceding germination is often constrained [48].

### 4.2. Wildflower Pollinators Attractivity and Biodiversity

The ecosystem service provided from the fifteen wildflower species, namely the provision of pollen and nectar during the warmer summer periods, was fully achieved. All tested species exhibited full flowering not only in May, a period characterized by high availability of alternative floral resources, but also in June and July, when the Mediterranean environment typically experiences a marked scarcity of herbaceous [49] and shrub blooms [50]. This result is particularly relevant in the context of climate change, which is known to induce earlier flowering phenology [51]. More than half of the species continued flowering into August, an even more critical period for pollinator food availability, and some extended their flowering period into September and October. Among these, three species belonging to the Dipsacaceae family (*C. transsylvanica*, *S. columbaria*, and *S. ochroleuca*) showed particularly high suitability, confirming previous findings for *C. transsylvanica* [52]. A similar tendency toward prolonged flowering was also observed in two of the three tested Malvaceae species (*A. cannabina* and *M. sylvestris*) as well as in *D. carota*.

From a quantitative perspective, June and July, the only months during which all fifteen wildflower species reached the flowering phenological stage, recorded the highest pollinator visitation rates. However, pollinator activity observed in subsequent months, although reduced, should not be underestimated. The extended flowering period of several species highlighted their superior performance in providing ecosystem services during the summer. In particular, Dipsacaceae, Malvaceae, and *D. carota* exhibited the highest cumulative visitation rates across the entire summer period.

The main visitors were Apoidea (honey bees, solitary bees, and bumblebees), which accounted for nearly 80% of pollinator diversity. Although honey bees and solitary bees were generally the most abundant Apoidea groups, bumblebees predominated in some cases, accounting alone for approximately 40% of the total visits recorded during the experimental period. This was observed for *S. germanica* and *V. sinuatum*. A similar, though less pronounced, dominance of bumblebees (about 30%) was also recorded for *C. regalis*, *C. varia*, and *L. punctata*.

This preference for bumblebees appears to be associated with the zygomorphic symmetry of the flowers [53] as well as with the long tongues of many bumblebee species, which allow them to reach nectaries located at the base of elongated corollas [54]. Consequently, these wildflower species may play a crucial role in the conservation of bumblebees, which are increasingly threatened by biodiversity loss driven by ongoing climate change [55].

Similarly, *D. carthusianorum*, belonging to the botanical family Caryophyllaceae, showed a marked specialization for Lepidoptera. This specialization, which is common within Caryophyllaceae, appears to be linked to floral scent composition [56], nectar chemistry [57] and the narrow, elongated corolla shape, which makes nectaries accessible to the long proboscis typical of most Lepidoptera [58]. The high abundance of lepidopteran visits observed for some of the tested species highlights the strong evolutionary relationships between wildflower and pollinator taxa.

Indeed, the presence of species that have primarily evolved for specialized pollination, as in this case, may explain why the attractiveness of wildflowers to bees and hoverflies is strongly species-dependent [59]. Supporting this hypothesis, studies conducted in European agroecosystems have shown that in some wildflower strips the majority of flower visitors were neither bees nor hoverflies [60,61]. Although wildflower–pollinator associations are often linked to botanical family-level traits, largely mediated by floral chemistry [62], this pattern was not confirmed in the present study. Instead, the observed relationships appear to be driven primarily by individual plant species. In fact, species belonging to the botanical families Malvaceae, Asteraceae, and Dipsacaceae exhibited varying degrees of generalization or specialization, even among species within the same family.

In quantitative terms, Dipsacaceae (notably *C. transylvanica* and *S. columbaria*), *Daucus carota* (Apiaceae), and *Malva sylvestris* (Malvaceae) exhibited the highest levels of pollinator attractiveness. This pattern of generalist or specialized attractiveness of the above-mentioned species was widely observed, thereby supporting a satisfactory level of pollinator taxonomic biodiversity. The above-mentioned Dipsacaceae displayed not only high attractiveness but, above all, an excellent ecosystem service by providing both pollen and nectar, particularly during the warmest and driest months (July and August). These findings confirm the previously reported outstanding performance of *C. transylvanica* and further highlight a strong summer-flowering aptitude for the other Dipsacaceae species, *S. columbaria* and *S. ochroleuca*.

Shannon biodiversity indices (H′), reflecting the complexity of pollinator assemblages visiting each wildflower species, showed the highest values for generalist species, in agreement with similar studies [63]. Specifically, the highest H′ values (>0.7) were recorded for *A. radiatus*, *D. carota*, *H. perforatum*, *S. columbaria*, and *V. sinuatum*, species that did not exhibit specialization toward particular insect pollinator taxa. In contrast, the two species with zygomorphic flowers (*C. regalis* and *S. germanica*), as well as *D. cartusianorum*, showed a higher degree of pollinator specialization, as indicated by lower H′ values.

### 4.3. Wildflowers Sustainability and Functionality over Time

Unfortunately, in the second year, the abundance of the fifteen wildflower species declined. Although this outcome was expected, it is noteworthy that the tested species exhibited markedly different levels of survival (for perennials) or self-seeding capacity (for annuals). Overall, perennial species such as *A. cannabina*, *C. varia*, *D. carota*, and *S. columbaria* showed the highest survival rates. This pattern appears to be related to the fact that, for annual species, germination ecology represents a limiting factor, particularly in compacted soils rich in plant residues with allelopathic activity, thus highlighting the poor sustainability of the wildflower strip over time [64]. Accordingly, annual species such as *A. radiatus*, *C. regalis*, and *L. punctata* exhibited the greatest declines. *C. transylvanica* represents an exception, likely due to its lower sensitivity to germination-related ecological constraints (e.g., soil softness and allelopathic inhibition from plant residues).

As expected, the reduction in flowering resulted in a decrease in visitation rates across species, although the magnitude of this decline varied considerably among them, while overall pollinator biodiversity remained unchanged. The highest performance in terms of sustained attractiveness was observed for *A. cannabina*, *C. varia*, *M. sylvestris*, and *S. columbaria*, as their visitation rates in the second year declined by less than 10% compared to the year of sowing.

## 5. Conclusions

The hypothesis that the selected species could meet pollinators’ requirements in terms of pollen and nectar availability during the hottest and driest periods was confirmed. However, germination and emergence capacity should not be underestimated, as seeds of wild species typically exhibit primary dormancy-removable through appropriate seed treatments-and may also develop secondary dormancy induced during seed burial after sowing [65], which can be agronomically managed through soil rolling.

Despite these constraints, several selected species showed promising performance in terms of wildflower strip establishment (seedling emergence rate), flowering dynamics, and pollinator attractiveness. As anticipated, wildflower strip performance in terms of flower abundance and pollinator attractiveness was highest during the first year after planting. Nevertheless, some species maintained satisfactory performance in the second year, indicating that future wildflower strips could be not only effective but also more sustainable over time.

Further research on these and other insect-pollinated species widespread in Mediterranean agroecosystems may support the refinement of this agronomic strategy, contributing to the conservation of pollinator biodiversity, which is increasingly threatened by ongoing climatic stresses.

## Figures and Tables

**Table 1:** Botanical and ecological information and seed collection environment of the fifteen species tested as summer wildflowers (H = hemicryptophyte, T = Terophyte). The 1000 seed weight values are followed by ± standard error of the means.

Wildflower Species	Botanic Family	1000 Seed Weight (g)	Environment of Seed Collection	Life Cycle
*Althaea cannabina* L.	Malvaceae	4.24 ± 0.32	Roadside	H
*Anacyclus radiatus* Loisel.	Asteraceae	0.12 ± 0.01	Crop edges	T
*Cephalaria transylvanica* (L.) Roem. and Schult.	Dipsacaceae	5.25 ± 0.43	Roadside	T
*Cichorium intybus* L.	Asteraceae	1.58 ± 0.14	Crop edges	H
*Consolida regalis* Gray	Ranunculaceae	1.52 ± 0.15	Crop edges	T
*Coronilla varia* L.	Fabaceae	4.58 ± 0.36	Roadside	H
*Daucus carota* L.	Apiaceae	1.22 ± 0.16	Arid grassland	H
*Dianthus carthusianorum* L.	Caryophyllaceae	1.35 ± 0.21	Grasslands	H
*Hypericum perforatum* L.	Hypericaceae	0.18 ± 0.02	Pastures	H
*Lavatera punctata* L.	Malvaceae	5.45 ± 0.42	Crop edges	T
*Malva sylvestris* L.	Malvaceae	3.98 ± 0.34	Crop edges	H
*Scabiosa columbaria* L.	Dipsacaceae	2.44 ± 0.22	Arid grassland	H
*Scabiosa ochroleuca* L.	Dipsacaceae	1.96 ± 0.18	Arid grassland	H
*Stachys germanica* L.	Lamiaceae	1.32 ± 0.12	Grasslands	H
*Verbascum sinuatum* L.	Scrophulariaceae	1.25 ± 0.01	Crop edges	H

**Table 2:** Germination of the fifteen wildflower species before and after seed treatment. Means (*n* = 4) are followed by ± standard errors. The statistical significance (^1^) is reported: * = significant for *p* < 0.05, ** = significant for *p* < 0.01 according to the Duncan’s Multiple Range Test.

Wildflower Species	Germination Before Seed Treatment %	Seed Treatment	Germination After Seed Treatment %	Statistical Significance ^1^
*Althaea cannabina*	23.2 ± 2.0	Scarification	76.5 ± 5.2	**
*Anacyclus radiatus*	37.5 ± 2.8	Washing	52.3 ± 3.8	*
*Cephalaria transsylvanica*	48.1 ± 4.2	Washing	75.6 ± 6.2	**
*Cichorium intybus*	35.6 ± 2.9	Washing	64.2 ± 5.6	**
*Consolida regalis*	15.5 ± 2.0	Chilling	54.5 ± 4.3	**
*Coronilla varia*	25.6 ± 2.2	Scarification	88.5 ± 7.3	**
*Daucus carota*	38.4 ± 3.6	Washing	65.4 ± 4.4	*
*Dianthus cartusianorum*	47.4 ± 3.8	Chilling	72.2 ± 6.2	**
*Hypericum perforatum*	28.7 ± 2.3	Chilling	41.4 ± 3.5	*
*Lavatera punctata*	21.4 ± 1.9	Scarification	88.2 ± 7.4	**
*Malva sylvestris*	24.6 ± 2.0	Scarification	92.2 ± 8.5	**
*Scabiosa columbaria*	35.7 ± 3.1	Washing	77.3 ± 6.1	**
*Scabiosa ochroleuca*	38.4 ± 3.4	Washing	75.5 ± 4.5	**
*Stachys germanica*	32.5 ± 2.8	Chilling	66.2 ± 5.8	**
*Verbascum sinuatum*	18.5 ± 1.6	Chilling	51.5 ± 2.8	*

**Table 3:** Seedling emergence % of treated seed (see Table 2) of the fifteen wildflower species. Means (*n* = 4) are followed by ± standard errors. The statistical significance (^1^) is reported: * = significant for *p* < 0.05, ** = significant for *p* < 0.01 according to Duncan’s Multiple Range Test.

Wildflower Species	Seedling Emergence %		Statistical Significance ^1^
	Sowing Without Soil Rolling	Soil Rolling After Sowing	
*Althaea cannabina*	46.5 ± 4.2	54.5 ± 3.2	*
*Anacyclus radiatus*	14.3 ± 3.8	35.6 ± 2.5	**
*Cephalaria transsylvanica*	55.6 ± 5.2	61.2 ± 5.3	*
*Cichorium intybus*	34.4 ± 3.6	45.2 ± 5.6	*
*Consolida regalis*	24.4 ± 2.2	35.2 ± 3.3	*
*Coronilla varia*	49.2 ± 4.3	58.3 ± 4.1	*
*Daucus carota*	28.2 ± 2.4	38.1 ± 3.0	*
*Dianthus cartusianorum*	35.2 ± 3.2	72.2 ± 6.2	**
*Hypericum perforatum*	22.1 ± 3.5	45.8 ± 3.2	**
*Lavatera punctata*	45.6 ± 4.4	55.0 ± 4.4	*
*Malva sylvestris*	53.7 ± 4.5	65.3 ± 5.5	*
*Scabiosa columbaria*	49.5 ± 4.1	58.5 ± 5.1	*
*Scabiosa ochroleuca*	45.3 ± 2.5	53.8 ± 4.2	*
*Stachys germanica*	22.2 ± 1.8	43.7 ± 3.7	**
*Verbascum sinuatum*	15.4 ± 1.2	38.3 ± 1.8	**

**Table 4:** Calendar of the flowering dynamics (● = flowering month of the studied species) of the tested wildflowers during the experimental periods.

Wildflower Species	Flowering Period (Months of the Year)											
	JAN	FEB	MAR	APR	MAY	JUN	JUL	AUG	SEP	OCT	NOV	DEC
*Althaea cannabina*					●	●	●	●	●	●		
*Anacyclus radiatus*					●	●	●					
*Cephalaria transsylvanica*					●	●	●	●	●			
*Cichorium intybus*					●	●	●	●				
*Consolida regalis*					●	●	●					
*Coronilla varia*					●	●	●					
*Daucus carota*					●	●	●	●	●			
*Dianthus cartusianorum*					●	●	●					
*Hypericum perforatum*					●	●	●					
*Lavatera punctata*					●	●	●					
*Malva sylvestris*					●	●	●	●	●	●		
*Scabiosa columbaria*					●	●	●	●	●			
*Scabiosa ochroleuca*					●	●	●	●	●			
*Stachys germanica*					●	●	●					
*Verbascum sinuatum*					●	●	●	●				

**Table 5:** Flower visit rate (number m^−2^ h^−2^) observed during each month of the blooming period (May–September) and total visitation rate (during all experimental period) detected in each of the fifteen wildflower species. Means (*n* = 10) are followed by ± standard errors. Means with the same letter within a row (flower visitation rate during the experimental months) or within a column (total visits rate) indicate no statistical difference (*p* < 0.05) according to the Duncan’s LSD test.

Wildflower Species	Flower Visitation Rate (Visits m^−2^ h^−1^)					Total Visitation Rate (Visits m^−2^ h^−2^)
	May	June	July	August	September	
*Althaea cannabina*	4.3 ± 0.1 a	24.5 ± 0.1 b	44.3 ± 0.1 c	50.2 ± 0.1 c	29.8 ± 0.1 b	153.1 ± 12.4 c
*Anacyclus radiatus*	18.3 ± 1.3 b	38.7 ± 2.9 c	28.4 ± 2.3 b	21.4 ± 2.1 b	10.8 ± 1.1 a	117.6 ± 10.1 a
*Cephalaria transsylvanica*	10.2 ± 1.1 a	43.5 ± 3.9 c	68.5 ± 5.0 c	75.5 ± 4.1 c	42.3 ± 4.0 b	240.0 ± 20.6 e
*Cichorium intybus*	5.5 ± 0.4 a	35.6 ± 2.9 c	55.3 ± 4.3 d	24.3 ± 2.1 c	18.4 ± 1.1 b	139.1 ± 12.8 b
*Consolida regalis*	22.4 ± 1.6 b	45.2 ± 2.9 c	41.6 ± 2.3 c	20.6 ± 2.1 b	5.5 ± 1.1 a	135.3 ± 10.1 b
*Coronilla varia*	10.5 ± 1.1 a	38.9 ± 3.2 c	44.5 ± 3.6 c	15.8 ± 1.6 b	4.0 ± 0.3 a	113.7 ± 11.4 a
*Daucus carota*	15.2 ± 1.2 a	28.6 ± 2.1 b	48.3 ± 3.8 c	43.5 ± 3.6 c	25.6 ± 2.2 b	161.2 ± 10.1 c
*Dianthus cartusianorum*	6.3 ± 0.4 a	35.6 ± 3.1 c	38.4 ± 3.3 c	22.3 ± 2.1 b	18.9 ± 1.5 b	121.5 ± 12.2 a
*Hypericum perforatum*	5.8 ± 0.3 a	38.3 ± 2.5 c	35.2 ± 2.9 c	15.8 ± 1.1 b	11.5 ± 0.3 a	115.3 ± 10.1 a
*Lavatera punctata*	4.4 ± 0.3 a	53.3 ± 3.9 c	50.2 ± 4.2 c	10.8 ± 1.8 b	4.9 ± 1.3 a	125.0 ± 12.6 a
*Malva sylvestris*	19.5 ± 1.5 a	43.2 ± 3.9 b	42.4 ± 3.7 b	36.6 ± 3.1 b	22.5 ± 2.1 a	164.2 ± 14.2 c
*Scabiosa columbaria*	12.2 ± 1.2 a	51.3 ± 3.6 c	55.2 ± 4.9 c	38.2 ± 2.5 b	30.8 ± 1.1 b	187.7 ± 16.6 d
*Scabiosa ochroleuca*	5.3 ± 0.4 a	40.4 ± 3.0 c	45.8 ± 3.6 c	42.3 ± 4.0 c	22.5 ± 2.1 b	156.3 ± 13.8 c
*Stachys germanica*	3.5 ± 0.3 a	60.5 ± 2.2 c	60.2 ± 4.3 b	19.2 ± 3.5 b	11.5 ± 1.0 b	155.0 ± 13.2 c
*Verbascum sinuatum*	4.0 ± 0.4 a	33.5 ± 3.1 c	35.4 ± 3.3 c	31.3 ± 2.8 c	18.3 ± 1.6 b	122.5 ± 11.4 a
**Means**	9.8 ±1.1 a	40.7 ± 3.9 d	46.2 ± 4.4 d	28.0 ± 2.7 c	18.7 ± 1.9 b	147.2 ± 12.8

**Table 6:** Flower visits percentages by different pollinator groups (Bees, Solitary bees, Bumblebees, *Diptera syrphydae*, *Diptera bombyliide*, *Diptera tachinidae*, Lepidoptera and Coleoptera) observed in each of the fifteen wildflower species. Means with the same letter within a row (flower visits percentage of the total) or within a column (total visitation rate) show did not differ for *p* < 0.05 according to the Duncan’s LSD test.

Wildflower Species	Flower Visits (% of the Total)							
	Honey Bees	Solitary Bees	Bumblebees	Diptera			Lepidoptera	Coleoptera
				Syrphidae	Bombyliidae	Tachinidae		
***Althaea cannabina***	**45.3 a**	**22.8 b**	**15.4 c**	**6.0 d**	3.7 e	0.2 e	4.5 d	2.1 e
*Anacyclus radiatus*	22.3 a	23.6 a	3.5 c	5.6 b	7.2 b	2.3 c	26.4 a	4.1 c
*Cephalaria transsylvanica*	35.2 b	41.5 a	12.3 c	3.3 d	2.1 d	1.2 d	4.2 d	0.2 e
*Cichorium intybus*	52.8 a	32.5 b	3.5 c	2.2 c	3.5 c	2.0 c	3.1 c	0.4 d
*Consolida regalis*	22.1 b	25.3 b	33.7 a	2.7 d	1.5 d	0 d	16.7 c	0 d
*Coronilla varia*	29.2 b	25.5 b	31.7 a	3.2 d	2.3 d	0 e	8.1 c	0 e
*Daucus carota*	45.7 a	18.9 b	4.2 c	4.6 c	3.4 d	5.6 c	2.4 d	15.2 b
*Dianthus cartusianorum*	10.7 c	31.7 b	0 e	13.2 c	4.6 d	0 e	44.3 a	5.5 d
*Hypericum perforatum*	25.3 b	41.2 a	14.4 c	6.6 d	9.5 c	3.1 d	6.9 d	3.0 e
*Lavatera punctata*	18.3 b	22.7 b	31.2 a	6.2 d	4.8 d	2.1	10.2 c	4.5 d
*Malva sylvestris*	30.7 a	32.8 a	15.2 b	2.2 c	3.1 c	0 d	15.3 b	0.7 d
*Scabiosa columbaria*	37.4 a	32.4 a	14.2 b	1.2 c	0.8 c	0.4 c	13.4 b	0.2 c
*Scabiosa ochroleuca*	31.2 a	25.3 b	13.1 c	14.2 c	1.1 d	0.5 d	14.2 c	0.4 d
*Stachys germanica*	25.4 b	29.3 b	40.4 a	8.3 d	0.3 e	0 e	16.1 c	0.2 e
*Verbascum sinuatum*	22.1 c	28.5 b	41.2 a	0 e	3.0 e	0 e	5.2 d	0 e
**Means**	30.2 a	28.7 a	18.2 b	5.3 d	2.4 d	1.1 e	11.7 c	2.4 d

**Table 7:** Biodiversity level (Shannon index, H′) of pollinator visits of the fifteen tested wildflower species. Values are followed by ± standard error. Means with the same letter show did not differ at *p* < 0.05 according to the Duncan’s LSD test.

Wildflower Species	Shannon Index (H′)
*Althaea cannabina*	0.65 ± 0.04 b
*Anacyclus radiatus*	0.74 ± 0.06 d
*Cephalaria transsylvanica*	0.60 ± 0.05 b
*Cichorium intybus*	0.53 ± 0.03 a
*Consolida regalis*	0.51 ± 0.05 b
*Coronilla varia*	0.65 ± 0.05 b
*Daucus carota*	0.72 ± 0.06 c
*Dianthus cartusianorum*	0.53 ± 0.07 e
*Hypericum perforatum*	0.77 ± 0.06 d
*Lavatera punctata*	0.67 ± 0.05 c
*Malva sylvestris*	0.61 ± 0.05 b
*Scabiosa columbaria*	0.71 ± 0.06 c
*Scabiosa ochroleuca*	0.65 ± 0.05 b
*Stachys germanica*	0.52 ± 0.05 b
*Verbascum sinuatum*	0.74 ± 0.06 d

**Table 8:** Plant density of the second experimental year, relative flower visits loss and pollinator biodiversity loss (^1^ as percentage to respect to the previous year). Means (*n* = 10) are followed by ± standard error. The statistical significance (of both: flower visits loss ^2^ and biodiversity (Shannon index, H′) loss ^3^ to respect to previous year) is reported: n.s. = no significant, * = significant for *p* < 0.05, ** = significant for *p* < 0.01.

Wildflower Species	Residual Plant Density ^1^ %	Flower Visits Loss ^1^ %	Statistical Significance ^2^	Pollinator Biodiversity Loss ^1^ %	Statistical Significance ^3^
*Althaea cannabina*	74.2 ± 6.5	14.6 ± 0.4	n.s.	0.02 ± 0.001	n.s.
*Anacyclus radiatus*	44.3 ± 3.8	66.2 ± 0.5	**	0.02 ± 0.002	n.s.
*Cephalaria transylvanica*	55.8 ± 4.5	25.8 ± 7.6	*	0.03 ± 0.002	n.s.
*Cichorium intybus*	75.4 ± 6.5	35.8 ± 5.8	*	0.02 ± 0.003	n.s.
*Consolida regalis*	24.0 ± 6.5	61.2 ± 4.5	**	0.01 ± 0.002	n.s.
*Coronilla varia*	68.9 ± 5.6	23.6 ± 0.3	n.s.	0.03 ± 0.003	n.s.
*Daucus carota*	87.5 ± 7.2	14.4 ± 1.2	n.s.	0.02 ± 0.002	n.s.
*Dianthus cartusianorum*	69.8 ± 5.9	18.8 ± 1.4	n.s.	0.02 ± 0.001	n.s.
*Hypericum perforatum*	54.2 ± 4.6	21.1 ± 2.2	*	0.01 ± 0.001	n.s.
*Lavatera punctata*	52.6 ± 4.4	55.8 ± 5.1	**	0.02 ± 0.002	n.s.
*Malva sylvestris*	92.2 ± 8.2	5.5 ± 0.4	n.s.	0.03 ± 0.003	n.s.
*Scabiosa columbaria*	84.8 ± 7.6	8.5 ± 0.6	n.s.	0.02 ± 0.001	n.s.
*Scabiosa ochroleuca*	67.3 ± 6.0	25.3 ± 1.3	*	0.03 ± 0.002	n.s.
*Stachys germanica*	44.2 ± 3.9	54.5 ± 4.0	*	0.01 ± 0.001	n.s.
*Verbascum sinuatum*	34.6 ± 2.5	78.5 ± 3.5	**	0.02 ± 0.001	n.s.

## Data Availability

The original contributions presented in this study are included in the article. Further inquiries can be directed to the corresponding author.
